# A Rare Cause of Cardiogenic Shock: A Case Report of Aortic Regurgitation due to Rupture of a Fibrous Strand Suspending a Tricuspid Aortic Valve

**DOI:** 10.1016/j.case.2021.07.006

**Published:** 2021-08-12

**Authors:** Bob Ophuis, Chris P.H. Lexis, Wouter G. Wieringa, Yvonne L. Douglas, Marco W. Willemsen, Jeroen W. op den Akker, Jan A. Krikken

**Affiliations:** a Department of Cardiology, University of Groningen, University Medical Center Groningen, Groningen, The Netherlands; b Department of Cardiothoracic Surgery, University of Groningen, University Medical Center Groningen, Groningen, The Netherlands; c Department of Radiology, University of Groningen, University Medical Center Groningen, Groningen, The Netherlands

**Keywords:** Acute aortic regurgitation, Fibrous strand rupture, Transesophageal echocardiography

## Abstract

• Fibrous strand rupture is a rare but possible cause of (acute) aortic regurgitation.
• Transesophageal echocardiography could clarify the mechanism of aortic regurgitation.
• Emergency cardiac surgery could be a salvage therapy in severe aortic regurgitation.

## Graphical abstract

**Figure:**
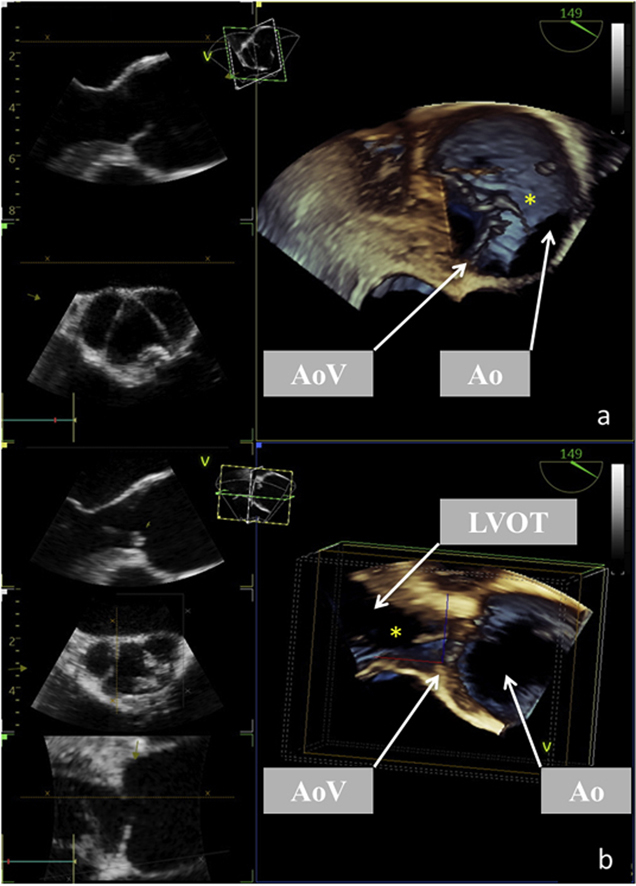

## Introduction

Acute aortic regurgitation (AR) caused by fibrous strand rupture is a very rare condition. Here we describe a case of acute AR resulting in severe cardiogenic shock caused by a ruptured fibrous strand originating at the aortic wall and supporting the structure of a tricuspid aortic valve.

## Case Presentation

**Figure 1:**
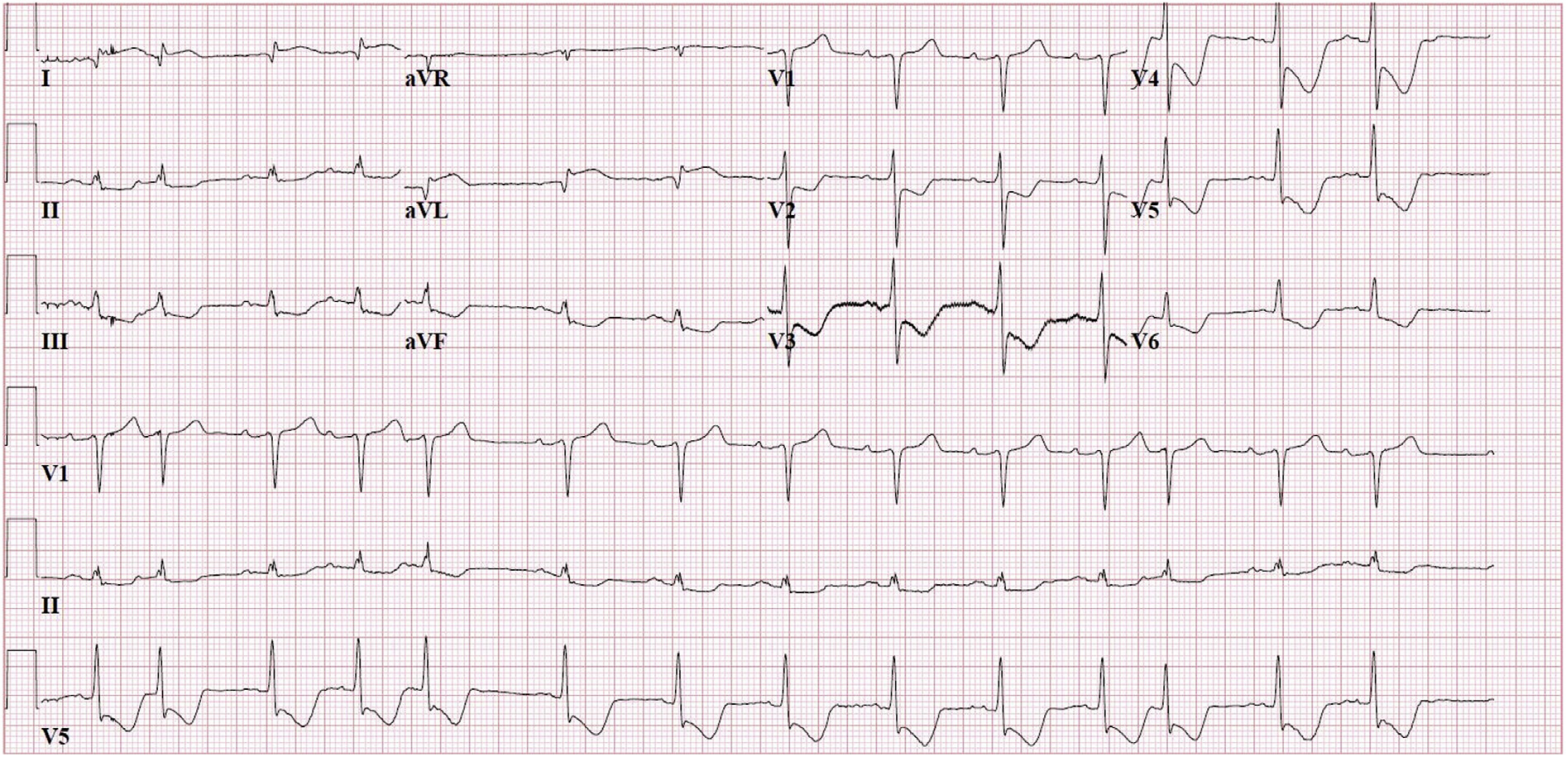
Electrocardiogram recorded at presentation. Sinus rhythm with frequent premature atrial complexes: ST-segment elevation is present in leads I and aVL, and ST-segment depression is present in leads II, III, aVF, and V2-V6.

A 73-year-old man presented at the emergency department because of a circulatory shock with sudden onset. His medical history revealed hypertension, a dilated sinus of Valsalva (47 mm), and ascending aorta (45 mm). Physical examination revealed a highly distressed and moaning patient in deep shock (blood pressure, 65/45 mm Hg; pulse, 100 bpm; lactic acid, 3.6 g/L). The electrocardiogram showed a pattern of global ischemia (Figure 1).

Transthoracic echocardiography (TTE) showed reduced left ventricular function (visually estimated left ventricular ejection fraction = 45%) and the previously established dilated aortic root. New findings included AR, which was difficult to quantify, and an abnormal configuration in the ascending aorta suggestive of an intimal flap. There was no pericardial effusion. The most likely diagnosis at this point was deemed a Stanford type A aortic dissection. However, emergency, electrocardiogram-triggered, computed tomography (CT) scanning excluded an aortic dissection. Moreover, there were no signs of central pulmonary embolisms and no signs of proximal coronary stenosis. However, a structure in the right coronary cusp (RCC) protruding into the aorta aimed at the aortic wall was seen. The exact significance of this finding was unclear at that time. A second TTE showed a situation comparable to that of the first TTE.

While the patient was still in severe cardiogenic shock, probably due to significant AR, but without a definite diagnosis, we decided to perform transesophageal echocardiography (TEE) in the operating room, taking into account the risk of further hemodynamic deterioration due to anesthesia and tracheal intubation.

As anticipated, the patient’s hemodynamics further deteriorated directly after tracheal intubation, necessitating resuscitation, which was successful. Meanwhile TEE was performed and revealed a tricuspid aortic valve, mild aortic root dilation (44 mm), and massive AR due to a flail of the RCC. The exact mechanism of the flail was not apparent at that moment, all the more because there were no signs of infective endocarditis. Despite the lack of a definite diagnosis, we decided for emergency sternotomy to try to perform an aortic valve replacement. The opened ascending aorta showed a tricuspid aortic valve and (the remnants of) a fibrous strand originating from the lateral side of the aortic wall, which presumably suspended the noncoronary cusp (NCC)/RCC commissure earlier.

The cardiothoracic surgeon replaced the insufficient aortic valve by a 23 mm mechanical aortic valve prosthesis. There was no need to replace the aortic root or ascending aorta. After a protracted intensive care unit stay, the patient recovered well and was ultimately discharged from the hospital 3 weeks after presentation.

**Figure 2:**
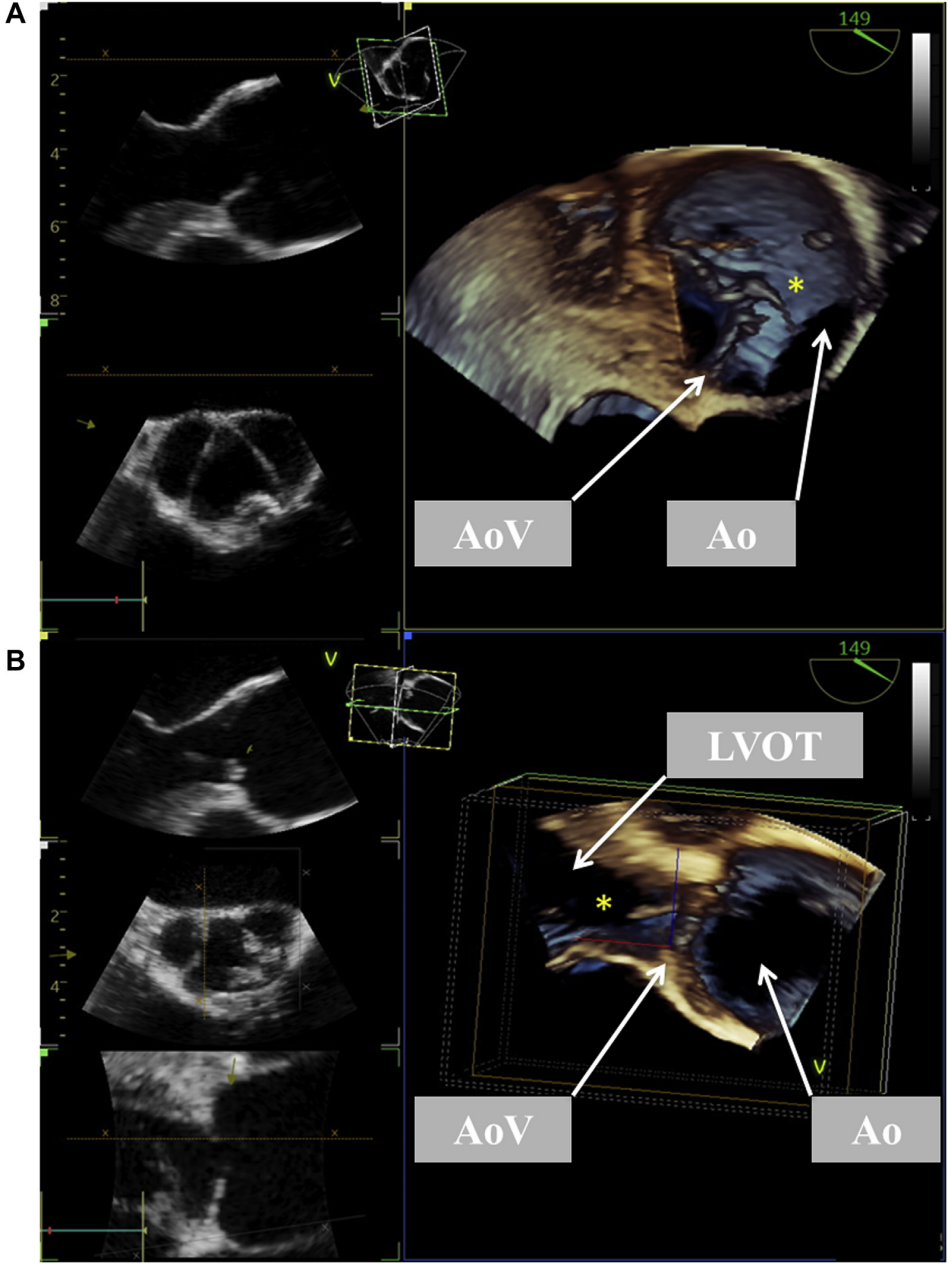
Three-dimensional TEE. **(A)** Systolic phase and **(B)** early diastolic phase showing the ruptured strand (∗) and flail of the RCC. *Ao*, Aorta; *AoV*, aortic valve; *LVOT*, left ventricular outflow tract.

**Figure 3:**
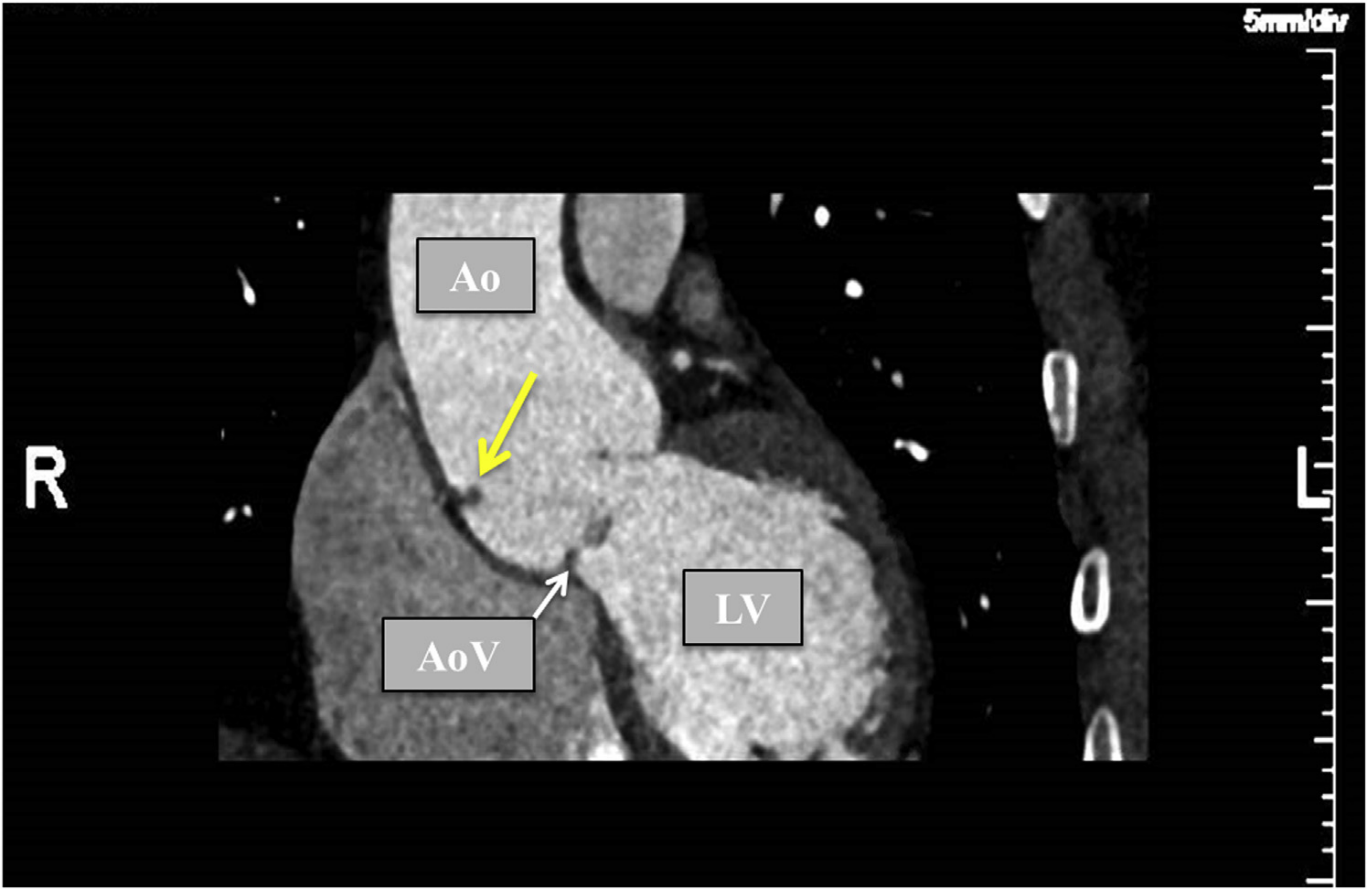
CT scan with arterial contrast enhancement showing the remnant of a fibrous strand at the free wall of the ascending aorta (*yellow arrow*). *Ao*, Aorta; *AoV*, aortic valve; *LV*, left ventricle.

**Figure 4:**
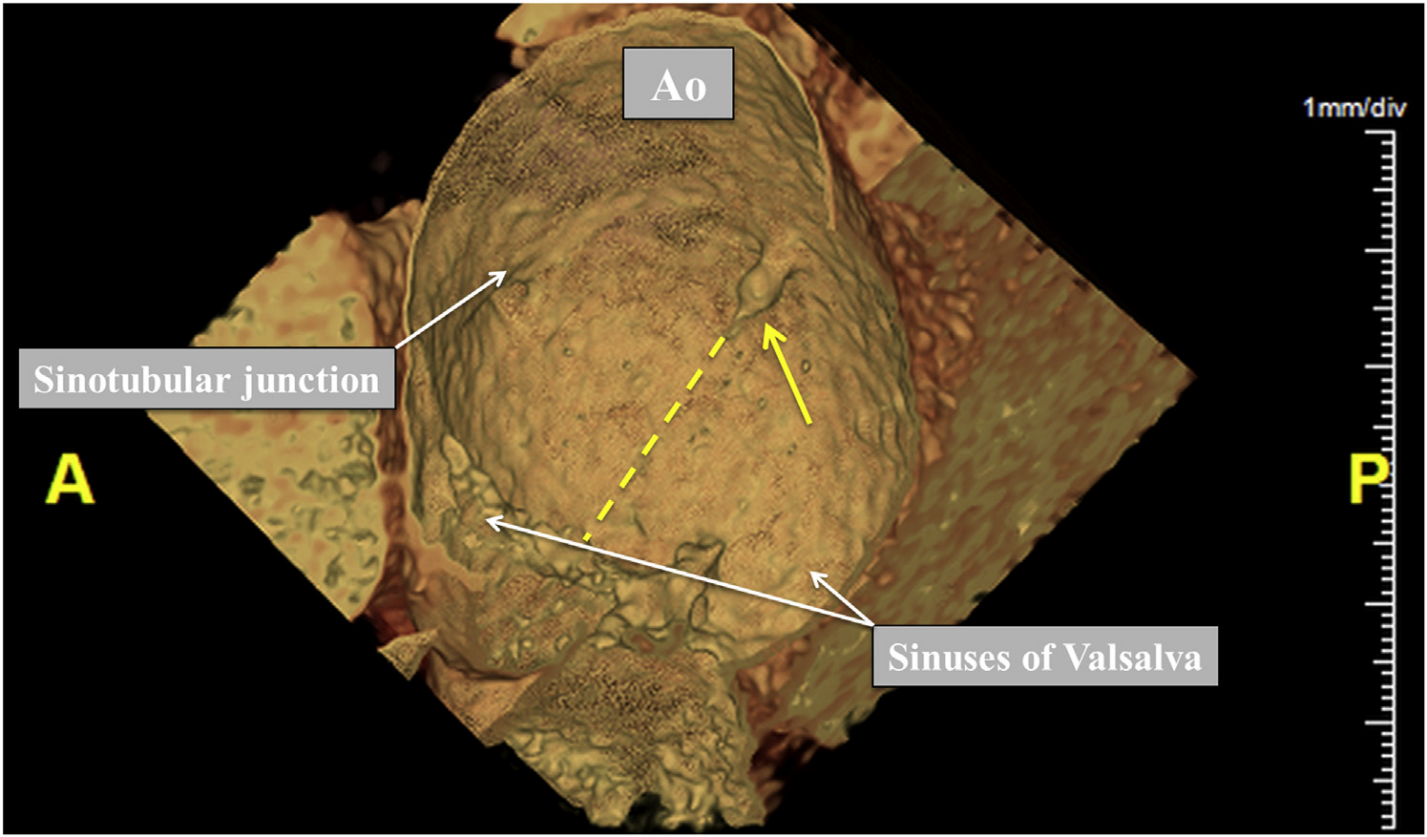
Three-dimensional CT scan showing the remnant of a fibrous strand (*yellow arrow*) and its former trajectory toward the aortic valve (*yellow dotted line*). *Ao*, Aorta.

Secondary analysis of the (three-dimensional) echocardiographic and CT scan images shows the unique anatomy of the aortic valve and the origin of the fibrous strand (Figure 2, Figure 3, Figure 4, Videos 1-5).

## Discussion

In this case, we hypothesize that a congenital fibrous strand, originating from the aortic wall and suspending the aortic valve apparatus, suddenly ruptured, resulting in acute AR. This rupture was probably triggered by progressive dilation of the sinus of Valsalva and ascending aorta, aggravated by hypertension. Clinical examination, echocardiography, blood cultures, and valve cultures ruled out infective endocarditis as the provoking factor.

**Figure 5:**
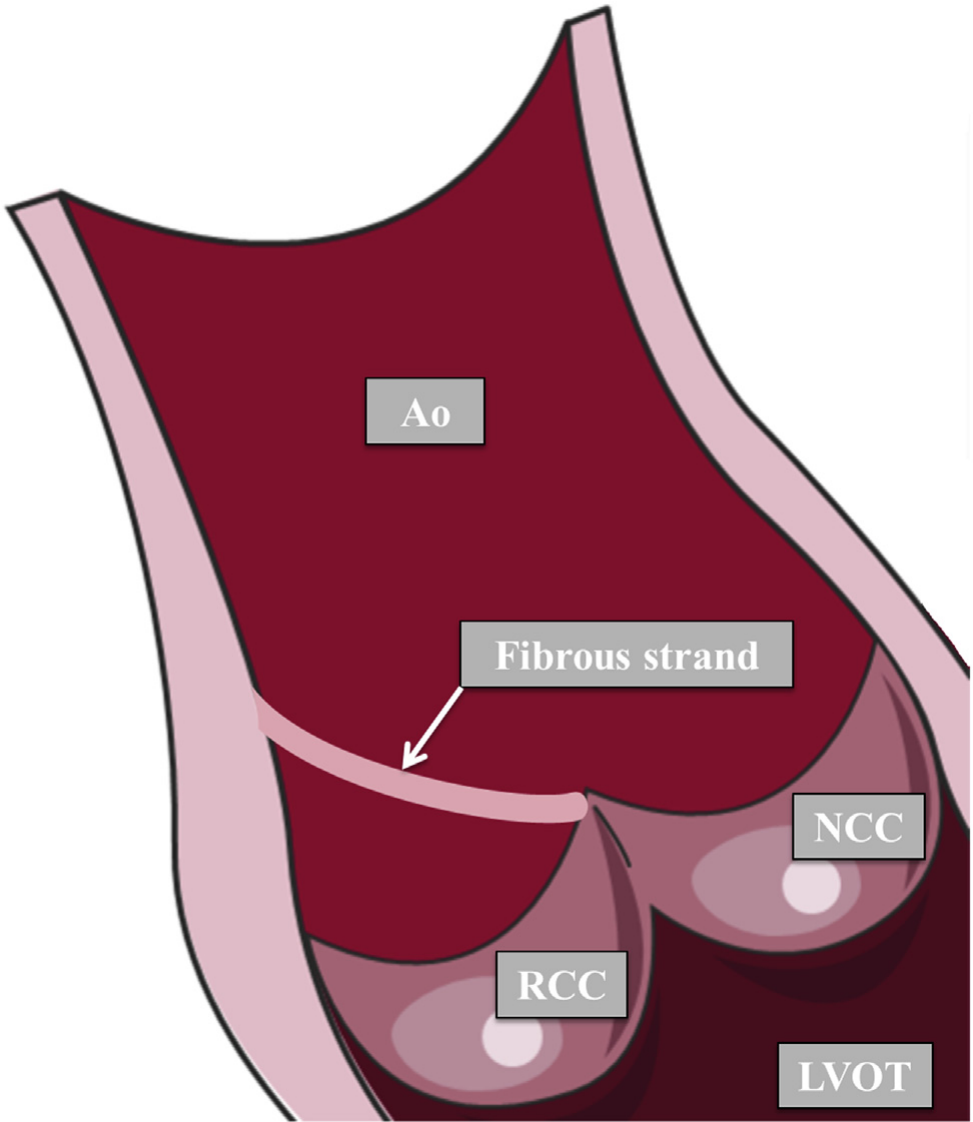
Schematic reconstruction of the fibrous strand and its trajectory. Originating at the aortic wall (sinotubular junction) and inserting at the NCC/RCC commissure of the aortic valve. *Ao*, Aorta; *LVOT*, left ventricular outflow tract. This figure was produced using images modified from Servier Medical Art (www.servier.com, licensed under a Creative Commons Attribution 3.0 Unported license).

The strand presumably had an essential supporting function in the aortic valve apparatus. The rupture led to an acute impairment of the NCC/RCC commissure, resulting in a flail of predominantly the RCC and acute AR followed by cardiogenic shock. This acute onset is also reflected by a relatively low N-terminal pro b-type natriuretic peptide (160 ng/L) at admission and the nondilated left ventricle at presentation. In a schematic reconstruction we give further insight into the supposed former trajectory of this fibrous strand (Figure 5).

In the majority of cases, acute AR is caused by either infective endocarditis of the aortic valve or an aortic dissection.^1^^,^^2^ In rare cases, acute AR is caused by a traumatic rupture of valve leaflets,^3^ due to complications of procedures such as aortic balloon valvotomy^4^ or rupture of fenestrated cusps.^5^

A few cases of AR in the presence of fibrous strands have been reported. The clinical course of these patients is variable, probably due to different mechanisms of AR development. Nonacute/chronic AR due to valve tenting and malcoaptation as a result of fibrous strands/aortic valve chordae tendinae has been described several times. Within these cases, the anatomy is highly variable. For instance, tricuspid aortic valves with single^6^ or multiple strands have been described,^7^, ^8^, ^9^ as well as a bicuspid valve with multiple strands.^10^ Few reports refer to AR as a consequence of a ruptured strand necessitating semielective^11^^,^^12^ or urgent surgical intervention.^13^

In our opinion, the clinical case we present in this report illustrates the importance of an integrated approach with different imaging modalities in a case of acute AR, especially when there is no clear-cut working diagnosis. When an acute Stanford type A aortic dissection is suspected, a CT scan is mandatory to confirm or exclude the diagnosis and guide further (surgical) management. Echocardiography provides helpful information regarding AR severity and mechanism, left ventricle function, and possible concomitant valvular disease. Furthermore, an alternative diagnosis, for instance, infective endocarditis, can be evaluated. In general TEE is not recommended when acute aortic dissection is suspected due to stress-induced hypertension possibly aggravating the (extent of) dissection. However, in anesthetized patients, TEE can be helpful, as our case illustrates, and in that case is superior to TTE. Magnetic resonance imaging may give comparable information to CT scanning but is time-consuming and rarely available and therefore not recommended in this setting. When the most likely causes of acute severe AR are excluded, our case stresses the importance of recognizing alternative rare diagnoses as a ruptured aortic strand. Careful consideration of all clinical information, even in an acute situation, will lead to the right diagnosis and prompt acute (salvage) intervention.

## Conclusion

Fibrous strand rupture is a rare cause of acute life-threatening AR. After ruling out more apparent causes of acute AR, recognition and subsequent acute intervention are required.

## Supplementary Data

Video 1 TEE, three-dimensional reconstruction showing the ruptured strand and flail of the RCC in an aortic view, anterior.

Video 2 TEE, three-dimensional sagittal plane reconstruction in RCC/left coronary cusp commissure, showing the ruptured strand and flail of the RCC.

Video 3 TEE, three-dimensional axial reconstruction of aortic root showing the flail of the RCC in a short-axis view.

Video 4 TEE, two-dimensional images showing the ruptured strand and flail of the RCC in a 0° five-chamber view.

Video 5 TEE, two-dimensional images showing the flail of the RCC in a 58° short-axis view.
